# The efficacy of edible film from Konjac glucomannan and saffron petal extract to improve shelf life of fresh‐cut cucumber

**DOI:** 10.1002/fsn3.1544

**Published:** 2020-05-27

**Authors:** Seyed Mohammad Bagher Hashemi, Dornoush Jafarpour

**Affiliations:** ^1^ Department of Food Science and Technology Faculty of Agriculture Fasa University Fasa Iran; ^2^ Department of Food Science and Technology College of Agriculture Islamic Azad University of Fasa Branch Fars Iran

**Keywords:** antimicrobial activity, antioxidant activity, cucumber, Konjac film, postharvest

## Abstract

The efficacy of saffron petal extract (SPE; 1%–4%) incorporated into Konjac glucomannan (KGM) edible films on the quality and shelf life of fresh‐cut cucumbers was evaluated. Changes in chemical, physical, and microbial properties, antioxidant activity, and total soluble phenolic contents of sliced cucumbers during storage at 4°C for 5 days were investigated. Results showed that the addition of SPE markedly reduced the water vapor permeability features of produced films, whereas the moisture content and transparency of them increased (*p* < .05). All the formulated films containing 1%–4% of SPE exhibited significant antimicrobial properties against the examined pathogens (*Escherichia coli*, *Shigella sonnei*, *Salmonella* Typhi, *Staphylococcus aureus,* and *Bacillus cereus*) both in vitro and in vivo conditions. KGM films incorporated SPE were successful in reducing mesophilic bacteria and fungi populations so that the microbial load significantly decreased as the concentrations of SPE increased and KGM + 4% of SPE was considered as the most effective treatment in decreasing the microbial content of sliced cucumbers. Total soluble solids of the treated cucumbers were significantly increased at the end of the storage in refrigerator, compared to the control sample. Moreover, antioxidant activity (DPPH assay) and total soluble phenols in treated fruit increased with storage time, while these parameters decreased with increasing concentrations of SPE incorporated into KGM film. So according to the findings, the introduced film with KGM and SPE could be considered as an edible film and be applied to preserve the fruit and vegetables quality and extend the shelf life of sliced cucumbers.

## Graphical abstract

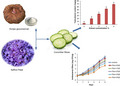

## 1 INTRODUCTION

Microbial contamination of foodstuffs plays an important role in consumer health. Adding chemical preservatives directly to the food is a common method of controlling microbial contamination. Due to the potential problems of synthetic preservatives on consumer health, consideration should be given to the use of natural, authorized, and generally recognized as safe (GRAS) preservatives (Gutierrez, Sanchez, Batlle, & Nerin, 2009; Hashemi, Niakousari, Saharkhiz, & Eskandari, 2014). The use of active packaging is a good way to protect food microbial without the direct addition of antimicrobial compounds to the food. Active packaging is a new type of packaging intended to improve the shelf life, safety, and sensory properties of foods. This type of packaging, along with the usual packaging roles such as physical protection, moisture, and gas inhibition, acts as a carrier of many active compounds such as antioxidants, antimicrobial, and flavoring agents (Hashemi, Zahabi, et al., 2016; Shojaee‐Aliabadi et al., 2013). Antimicrobial packaging is one of the types of active packaging in which antimicrobial compounds control microbial contamination by preventing the growth of spoilage and food poisoning microorganisms. On the other hand, in recent years, the increasing environmental damage caused by petroleum‐based plastics, the limitations of petroleum resources, as well as the technical problems of recycling plastic waste have led researchers and industry to pay more attention to find suitable alternatives to petroleum‐derived polymers (Coma, Freire, & Silvestre, 2015). Hence, biodegradable packaging based on natural polymers seems to be good alternatives to these substances and helps preserve the oil and the environment. Recent studies have shown that Konjac gum has the potential to produce film and can be used as a carbohydrate‐based biopolymer (Du et al., 2019; Saeheng, Eamsakulrat, Mekkerdchoo, & Borompichaichartkul, 2016). Konjac glucomannan (KGM), known as Konjac gum, is a polysaccharide derived from the tubers of an Asian plant called *Amorphophallus konjac*. It is a heteropolysaccharide containing d‐glucose and d‐mannose with beta (*β*) type junctions. KGM is water soluble, has high molecular weight, tends to form fine network when drying, is edible, and is used in food and pharmaceutical applications (Wang et al., 2017). Hongkulasap (2006) realized that apple coating with KGM solution (1%), glycerol (0.3% w/v), and 0.5 M KOH (0.14%) increased the edible quality of it compared to the uncoated sample. Although physical changes of fresh products can be well controlled, fresh products still face microbial deterioration problems. Extracts of plants and their constituents are natural compounds that have been known for their antimicrobial effects. Their numerous applications to control the growth of spoilage and foodborne pathogenic bacteria have led to their use as food preservatives (Canillac & Mourey, 2001; Hashemi, Amininezhad, Shirzadinezhad, Farahani, & Yousefabad, 2016). Saffron (*Crocus sativus* L.) is a small plant that belongs to the Iridaceae family. The dried stigma of this plant is used as a saffron in the food (as a fragrant spice for coloring food) and pharmaceutical (as a sedative and pain reliever for asthma, pertussis, and inflammation) industries (Okmen, Kardas, Bayrak, Arslan, & Cakar, 2016). In the process of saffron production, stigma is used as a commercial saffron, and other parts of the flower, including petals, are disposed every year in farms. Therefore, finding a solution to recycle and reuse these large amounts of waste is very important.

Hence, due to the useful properties of KGM to form films and the antimicrobial properties of saffron petals, there is a potential for application of antimicrobial and antioxidant edible films from KGM for fresh‐cut products to prevent postharvest damages from chemical, physical, and microbiological deterioration. Our study aimed to develop biodegradable edible KGM film added with extracts from useless and waste part of saffron (petal) to prevent postharvest losses of fresh‐cut cucumbers during storage.

## 2 MATERIALS AND METHODS

### 2.1 Materials and strains

KGM (food grade) with ≥95% purity was obtained from Pars Khooshe Pardaz Company. Saffron petals were prepared from saffron farms in Birjand city, Iran. Saffron petals were shade‐dried at room temperature and then powdered by kitchen grinder (Bosch, Germany). Cucumber (*Cucumis sativus*) was purchased from a fresh market in Fasa, Iran, and kept at 4°C until used. Analytical grade glycerol was supplied from Merck. Foodborne pathogenic bacteria, including the gram‐negative bacteria, *Escherichia coli* PTCC 1276, *Shigella sonnei* PTCC 1777, and *Salmonella* Typhi PTCC 1609, and the gram‐positive bacteria, *Staphylococcus aureus* PTCC 1764 and *Bacillus cereus* PTCC 1154, were provided by the culture collection at Iran Institute of Industrial and Scientific Research (Tehran, Iran). All the culture media and other chemical reagents were purchased from the Merck Company. All of the products were of analytical grade.

### 2.2 Plant material and extract

The alcoholic extracts of saffron petals were prepared according to the method of Sanchez‐Vioque et al. (2012) with slight modifications. 40 g of powdered petals was weighed and extracted by stirring in 400 ml of ethanol for 24 hr at ambient temperature. Suspensions were precipitated by centrifuging (Sigma) to remove sediments. After that, the supernatants were filtered (0.45 µm), and solvent (ethanol) was removed by placing in the vacuum drying oven at 45°C for 4 days. Finally, the dried extracts (with moisture content of 3.47% MC_wb_) were stored at −18°C until further analysis.

### 2.3 Preparation of Konjac film with SPE

For this purpose, the solution containing 6% of KGM and 25% (w/w) of glycerol was stirred and kept to 30 ± 1°C at 500 rpm for 20 min. Saffron petal extract (SPE) was added to film solution to get final concentration of 0, 1, 2, 3, and 4% (v/v). Finally, 20 ml of film‐forming solution was poured into Petri dishes and dried at 38°C for 48 hr.

### 2.4 Determination of physical properties of films

#### 2.4.1 Film thickness

Film thickness was measured with a digital micrometer (Mitutoyo No. 293‐766, Tokyo, Japan; ±0.001 mm). The average value of measurement of five various locations of films was used (Hashemi & Khaneghah, 2017).

#### 2.4.2 Moisture content

Moisture content of film pieces was calculated by weight loss determination of films, after drying in an oven at 90°C for 24 hr (Khazaei, Esmaiili, Djomeh, Ghasemlou, & Jouki, 2014).

#### 2.4.3 Transparency

For this test, after cutting the films into rectangular pieces (9 mm × 45 mm), they were placed on the internal side of spectrophotometer cell (UV/Visible Philips). The transmittance was recorded at 600 nm, and the film transparency was measured (Hashemi, Khaneghah, Ghahfarrokhi, & Eş, 2017).

#### 2.4.4 Water vapor permeability

The film samples were sealed in glass permeation cups containing silica gel, and the cells were kept in desiccator with distilled water at 30°C. The cups were weighed at an interval of 1 hr through 24 hr, and water vapor permeability (g m^−1^ s^−1^ Pa^−1^) was calculated (Siripatrawan & Harte, 2010).

### 2.5 In vitro antibacterial activity of edible film

*Escherichia coli* PTCC 1276, *Shigella sonnei* PTCC 1777, *Salmonella* Typhi PTCC 1609, *Staphylococcus aureus* PTCC 1764, and *Bacillus cereus* PTCC 1154 were used to evaluate the antibacterial properties of the films. These strains were purchased and reactivated in Nutrient Broth (Oxoid, UK) at 35–37°C. For antibacterial activity test, a suspension (0.1 ml of 10^6^ CFU/ml) of each strain was spread on the Mueller–Hinton plates. The film disks (10 mm diameter) were aseptically placed on the inoculated plates. Then, the plates were incubated at 35–37°C through 24 hr and the diameters of the inhibition zones (mm) around the films were measured with caliper (Sayanjali, Ghanbarzadeh, & Ghiassifar, 2011).

### 2.6 Total phenolics content of film samples

Approximately, 25 mg of each film sample was dissolved in 3 ml of distilled water and 0.3 ml of film extract was blended with 8 ml of distilled water and 0.6 ml of Folin–Ciocalteu reagent. After incubation for 10 min at ambient temperature, 0.1 ml of distilled water and 1.5 ml of sodium carbonate solution were added. The solution was kept for 2 hr, and then, the absorbance was read at 765 nm (Siripatrawan & Harte, 2010).

### 2.7 DPPH radical scavenging activity of film samples

For this test, 25 mg of each film was dissolved in 3 ml of methanol, and afterward, 2.7 ml of film extract solution was blended with 0.3 ml of DPPH in methanol (1 mM). The solution was shaken and subsequently placed in a dark place. Absorbance at 517 nm was read following 1 hr for all samples (Kam et al., 2018).

### 2.8 Treatment of fresh‐cut cucumber with the films and storage

Before treatment, cucumbers with consistent size and form with no signs of mechanical damage and fungal decay were chosen and washed with tap water followed by air‐dried at room temperature. After that, samples were arbitrarily divided into six different treatments (control: with no film and film with 0%, 1%, 2%, 3%, and 4% extract). Cucumbers were manually cut into slices with a sharp stainless‐steel knife and then coated with film solution. Samples were placed in polystyrene plastic tray (1,000 ml) covered by a lid and sealed with parafilm. Then, samples were kept at 4°C for up to 5 days at a relative humidity of 80%–90%. Every day, samples were taken for assessment of the chemical properties and microbial growth.

### 2.9 In vivo antimicrobial activity of the treated films

Briefly, 100 μl of each pathogen (*E. coli*, *S. sonnei*, *S.* Typhi, *S. aureus*, and *B. cereus*) resulting to 6.1 log CFU/ml separately was spotted on the surface of cucumber slices. Afterward, the samples were air‐dried at 25°C for 2 hr before treatments to allow pathogen attachment. After inoculation, samples were coated with various film solutions (control: with no film and film with 0%, 1%, 2%, 3%, and 4% extract). Treated samples were kept at 4°C for up to 5 days, and at the end of storage, microbial analyses were carried out. For microbial analyses, each sample was mixed with peptone water in a stomacher (BagMixer 400 W, Interscience Co.) for 2 min, and after serial dilution, plating was performed onto proper medium. Finally, incubation was done at 35–37°C for 24 hr.

### 2.10 Total plate count (TPC) and yeast/mold count (YMC) changes of fresh‐cut cucumbers

For TPC and YMC analysis, each sample was transferred into a sterile stomacher bag containing 0.1% peptone water and homogenized for 5 min with a stomacher. Afterward, serial dilutions at proper diluents were carried out. Each dilution was plated in an appropriate medium. Nutrient agar and Yeast Glucose Chloramphenicol Agar were used as a proper medium for aerobic bacteria and fungi (yeast and mold), respectively (Hashemi et al., 2017).

### 2.11 Total soluble solids of samples

After homogenization of cucumber slices, samples were filtered and total soluble solids (%) were calculated by a digital refractometer (DR‐301‐95).

### 2.12 Total soluble phenolics

A 50 µl of filtrated cucumber extract from a blend of 6 cucumber slices in each treatment was diluted by water (800 µl) and 0.2 N Folin–Ciocalteu reagent (25 µl). Then, incubation was performed for 5 min. Subsequently, 100 µl of sodium carbonate was mixed with the solution and incubation was done at ambient temperature for 1 hr. Absorbance of the solution was read at 750 nm (Peretto et al., 2014).

### 2.13 DPPH radical scavenging activity of samples

A 5 g of cucumber slices was extracted with methanol (15 ml). After that, the solution was centrifuged for 15 min at 5,000 g and 150 µl of obtained supernatant was added to 2,850 µl of DPPH reagent (0.047 g/L) in methanol. After storing for 24 hr, the absorbance was read at 517 nm (Peretto et al., 2014).

### 2.14 Statistical analysis

Statistical test was performed using the SPSS program (SPSS 23.0 for window, SPSS Inc.). Analysis of variance (ANOVA) was used for statistical analysis, and the differences between means were carried out by Duncan's multiple range tests. Level of significance was set for *p* < .05.

## 3 RESULTS AND DISCUSSION

### 3.1 Physical properties of KGM films containing SPE

The effects of mixing SPE on the physical properties of the KGM film are shown in Table 1. According to the results, it was found that by increasing the concentration of SPE, the thickness of KGM films did not change significantly (*p* > .05) and the thickness of the films was recorded as 0.07 mm. The thickness of the films depends on the concentration of the ingredients, the amount of initial solution of the film, and the rate of pouring on the surface. Our results are similar to Bitencourt, Fávaro‐Trindade, Sobral, and Carvalho (2014) findings. They found that incorporation of curcuma ethanol extract to gelatin‐based film did not alter the thickness of the film. The moisture content of KGM film was significantly increased (*p* < .05) from 16.61% to 17.17% as SPE content enhanced from 1% to 4%. Aguirre, Borneo, and León (2013) reported that incorporation of oregano oil at 2% remarkably affects the moisture content of triticale protein films. The transparency of films used in the packaging has a direct effect on the product appearance and consumer acceptance. As can be seen in Table 1, the transparency of the pure KGM film was recorded as 1.94. Subsequently, data showed that by incorporation of SPE from 1% to 4% to the films, the clearness notably increased (*p* < .05) from 2.44 to 3.88. It seems that due to the transparent and clear nature of the applied extracts, by increasing the percentage of SPE, the light permeability is increased and the produced films become more transparent. Our result is consistent with the findings of Wu et al. (2013), which adds green tea extract into an active film from silver carp (*Hypophthalmichthys molitrix*) skin gelatin, and found that increasing green tea extract concentrates increased the transparency of gelatin films. On the other hand, these results are in contrast to Benavides, Villalobos‐Carvajal, and Reyes (2012) findings. They observed that as the concentration of essential oil increased, the transparency of alginate films decreased. Water vapor permeability (WVP) is a significant indicator, since films used in food packaging are necessary to reduce water permeability. The film permeability to water vapor is directly related to the hydrophilic nature of the film, in which the hydrophilic film has a higher WVP and implies less inhibition toward water vapor (Wang et al., 2014). WVP of the tested films was investigated in different treatments. According to the results (Table 1), WVP of the pure KGM film was 4.86 × 10^−11 ^g H_2_O m^−1^ s^−1^ Pa^−1^ which was markedly decreased (*p* < .05) by the addition of SPEs into the KGM film matrix (4.67 × 10^−11^ and 3.95 × 10^−11^ g H_2_O m^−1^ s^−1^ Pa^−1^ for 1% and 4% of SPE incorporation, respectively). Studies have shown that KGM films are sensitive to moisture and have poor WVP properties due to their hydrophilic character (Wu et al., 2018). It seems that increasing the amount of SPE might form a network with reduction in free volume of the KGM matrix, leading to permeability reduction of the resulting film. Similarly, Rattaya, Benjakul, and Prodpran (2009) showed that moisture barrier's ability of fish skin gelatin film improved by formulating with seaweed extract.

**TABLE 1:** Properties of Konjac glucomannan film enriched with different concentrations of saffron petal extract

Concentration (V/V; %)	Film properties			
	Thickness (mm)	Moisture content (%)	Transparency	Water vapor permeability (×10^−11^g/ms Pa)
0	0.07 ± 0^a^	16.41 ± 0.07^e^	1.94 ± 0.03^e^	4.86 ± 0.7^a^
1	0.07 ± 0^a^	16.61 ± 0.05^d^	2.44 ± 0.06^d^	4.67 ± 0.10^b^
2	0.07 ± 0^a^	16.84 ± 0.08^c^	2.91 ± 0.05^c^	4.41 ± 0.05^c^
3	0.07 ± 0^a^	16.97 ± 0.07^b^	3.46 ± 0.09^b^	4.18 ± 0.06^d^
4	0.07 ± 0^a^	17.17 ± 0.06^a^	3.88 ± 0.11^a^	3.95 ± 0.05^e^

Note

Values represent means ± *SD*. Means within a column with the same lowercase letters are not significantly different at *p* < .05.

### 3.2 Antimicrobial activity of KGM film containing SPE

The antimicrobial activities of KGM films enriched with different concentrations of SPE (1%–4%), against five tested bacterial strains, are illustrated in Table 2. The pure KGM film was not effective against both gram‐negative and gram‐positive bacteria. Furthermore, similar findings were reported by the previous studies regarding the antibacterial characteristics of formulated films by gelatin (Martucci, Espinosa, & Ruseckaite, 2015) and zein (Moradi, Tajik, Rohani, & Mahmoudian, 2016). On the contrary, a considerable antimicrobial inhibition against target bacteria was recognized with higher SPE concentrations that confirm the direct correlation between antimicrobial properties and percentage of used extracts (*p* < .05). Among tested bacteria, gram‐positive bacteria (*S. aureus* and *B. cereus*) were more sensible against KGM film containing SPE with an inhibitory zone of 18.5–34.3 and 16.3 –32.4 mm^2^. Data showed that by application of SPE in concentration of 4%, a greater antibacterial activity was evaluated in the case of *S. aureus* followed by *B. cereus*, *S. sonnei*, *E. coli,* and *S.* Typhi with a clear zone of 34.3, 32.4, 29.8, 27.5, and 27.8 mm^2^, respectively. Our results are in synchronization with the report of Pelissari, Grossmann, Yamashita, and Pineda (2009). Similar antibacterial efficacy was also recorded by Shojaee‐Aliabadi et al. (2013) for *Satureja hortensis* essential oil by incorporation in film from κ‐carrageenan which revealed that gram‐positive bacteria are more sensitive in comparison with gram‐negative bacteria. There have been several studies on the inhibitory effect of saffron extract; however, the antibacterial activities of SPE incorporated into KGM film have not been investigated yet (vahidi, Kamalinejad, & Sedaghati, 2002). It seems that the presence of active compounds such as safranal and crocin in extracts of saffron petals could be responsible for the bactericidal activity observed (Pintado et al., 2011). Effeciency of SPE against gram‐positive bacteria was better than gram‐negative bacteria probably due to the action of extracts and owing to lack of lipopolysaccharide in gram‐positive bacteria that may be an effective barrier against any incoming biomolecule. Moreover, intrinsic resistance against toxic components in gram‐negative bacteria causes a permeability barrier in the outer cell envelope against toxic factors (Delaquis, Stanich, Girard, & Mazza, 2002).

**TABLE 2:** Antibacterial activity of Konjac glucomannan film enriched with various concentrations of saffron petal extract

Films	Diameter (mean and *SD*) of inhibition zone (mm) including film (10 mm)				
	*S. typhi*	*E. coli*	*S. sonnei*	*B. cereus*	*S. aureus*
Control^a^	**–**	**–**	**–**	**–**	–
Film + 1%	13.3 ± 0.2^dD^	13.4 ± 0.3^dD^	14.8 ± 0.2^dC^	16.3 ± 0.3^dB^	18.5 ± 0.4^dA^
Film + 2%	17.8 ± 0.1^cD^	17.4 ± 0.2^cD^	19.3 ± 0.5^cC^	21.7 ± 0.4^cB^	24.8 ± 0.7^cA^
Film + 3%	23.6 ± 0.5^bD^	23.9 ± 0.6^bD^	25.6 ± 0.7^bC^	27.7 ± 0.4^bB^	29.9 ± 0.5^bA^
Film + 4%	27.8 ± 0.6^aD^	27.5 ± 0.4^aD^	29.8 ± 0.3^aC^	32.4 ± 0.7^aB^	34.3 ± 0.6^aA^

Note

Values represent means ± *SD* of inhibition zones. Means within a column with the same lowercase letters are not significantly different at *p* < .05, and means within a row with the same uppercase letters are not significantly different at *p* < .05.

^a^ Control: Film + 0% of SPE.

### 3.3 Antioxidant activity of KGM film containing SPE

**FIGURE 1:**
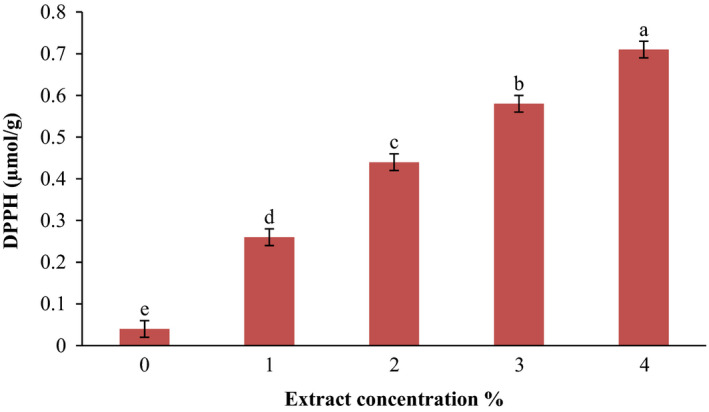
DPPH radical scavenging activity of film at different extract concentrations. Values represent means ± standard deviations. Each column with the same lowercase letters is not significantly different (*p* > .05)

**FIGURE 2:**
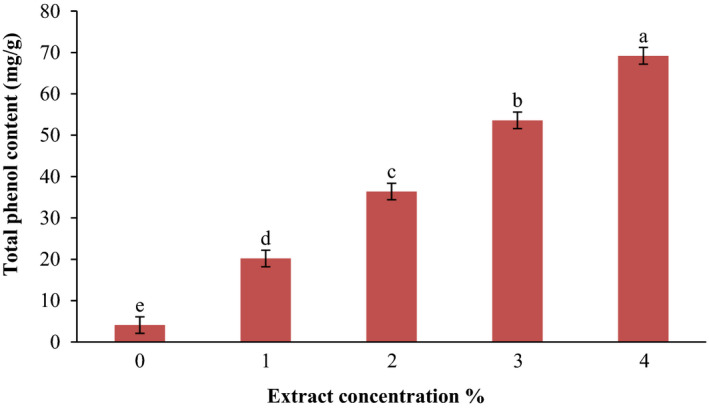
Total phenol content of film at different extract concentrations. Values represent means ± standard deviations. Each column with the same lowercase letters is not significantly different (*p* > .05)

In this study, DPPH radical scavenging activity and the total phenolics content were examined to evaluate the antioxidant activity of KGM film containing SPE since for specifying in vitro antioxidant activities of foodstuffs more than one assay is needed. DPPH radical scavenging activity of KGM film containing SPE is shown in Figure 1. As can be seen in the figure, pure KGM film had little inhibition activity, but with the addition of the extract, films represent more inhibitory effect. In addition, scavenging activity was significantly increased (0.26–0.71 µmol/g of the film) by the incorporation of SPE from 1%–4% (*p* < .05). Hafsa et al., (2016) reported that chitosan film with *Eucalyptus globulus* essential oil had radical scavenging property. Radical scavenging activity of saffron petals has been reported (Sariri et al.,^2011^). According to the data, it can be said that petal extract induces its inhibition effect to the KGM films against free radicals of DPPH. The same results were also achieved for total phenol content of KGM films with and without SPE (Figure 2). As the concentration of the SPE increased, the total phenol content of films also significantly increased (*p* < .05). The highest total phenol amount was recorded in 4% of SPE with 69.2 mg/g, and the lowest total phenol content was in pure KGM film with 4.1 mg/g. Researches have claimed that the phenolic compounds present in plants act as radical scavengers and the antioxidant capacity of the plants is mainly related to the presence of them. Also, it has been shown that phenolics can play an important role in the antimicrobial and antioxidant activities (Skerget et al., 2005). Noshirvani, Ghanbarzadeh, Rezaei Mokarram, and Hashemi (2018) showed that cinnamon essential oil had high oxidation activity and its addition to the chitosan‐carboxymethyl cellulose‐oleic acid‐based films produced desirable antioxidant films, which was a good characteristic in food packaging to protect nutrients from oxidation.

### 3.4 Survival of inoculated bacteria in fresh‐cut cucumbers

After examining the antimicrobial properties of KGM film containing SPE in vitro, the effect of edible coatings was also investigated in vivo condition. As shown in Table 3, the counts of all pathogenic bacteria significantly increased (*p* < .05) in control (without coating) and KGM (coating without SPE) samples after 5 days of refrigerated storage compared to initial inoculation (6.1 log CFU/g). Results showed that the use of KGM coatings with SPE reduced significantly (*p* < .05) the pathogenic bacteria levels in fresh‐cut cucumbers as compared to cucumber slices without coating and coated without incorporation of SPE. Also, it can be observed that as the concentration of SPE increased, the microbial counts significantly decreased (*p* < .05). The highest antimicrobial activity was illustrated by application of 4% of SPE in the case of *S. aureus* followed by *B. cereus*, *S. sonnei*, *S.* Typhi, and *E. coli* with 3.4, 3.5, 3.9, 4.4, and 4.5 log CFU/g, respectively, compared to initial inoculation (6.1 log CFU/g). In the study of Rojas‐Grau et al. (2007), the antimicrobial effect of essential oils against *Listeria innocua* inoculated into apple pieces was examined. They reported that Lemongrass (1.0 and 1.5% w/w) and oregano oil containing coatings (0.5% w/w) exhibited the strongest antimicrobial activity against *L. innocua* (4 log reduction). As observed in vitro, the extract used in the coating of cucumber slices has a greater effect on gram‐positive bacteria than gram‐negative bacteria which is due to the differences in bacterial cell wall.

**TABLE 3:** Antimicrobial activity of Konjac glucomannan edible coating enriched with various concentrations of saffron petal extract on the microbial counts of bacteria inoculated on cucumber slices (log CFU/g of fruit) during storage

	Initial count	Microbial count after 5 days				
		*S. typhi*	*E. coli*	*S. sonnei*	*B. cereus*	*S. aureus*
Control^a^	6.1 ± 0.02^aB^	7.8 ± 0.02^aA^	7.9 ± 0.05^aA^	8.0 ± 0.02^aA^	8.2 ± 0.03^aA^	7.9 ± 0.04^aA^
Film + 0%E	6.1 ± 0.01^aB^	7.4 ± 0.02^aA^	7.5 ± 0.03^aA^	7.6 ± 0.05^aA^	7.7 ± 0.03^aA^	7.4 ± 0.02^aA^
Film + 1%E	6.1 ± 0.01^aA^	5.8 ± 0.04^bAB^	5.6 ± 0.04^bB^	5.2 ± 0.04^bB^	4.8 ± 0.01^bC^	4.7 ± 0.01^bC^
Film + 2%E	6.1 ± 0.01^aA^	5.3 ± 0.02^bAB^	5.1 ± 0.02^bB^	4.9 ± 0.01^bB^	4.5 ± 0.02^bC^	4.3 ± 0.01^bC^
Film + 3%E	6.1 ± 0.02^aA^	4.8 ± 0.01^cB^	4.6 ± 0.02^cB^	4.1 ± 0.03^cC^	3.9 ± 0.02^cC^	3.8 ± 0.03^cC^
Film + 4%E	6.1 ± 0.01^aA^	4.4 ± 0.03^cB^	4.5 ± 0.01^cB^	3.9 ± 0.01^cC^	3.5 ± 0.04^cC^	3.4 ± 0.02^cC^

Note

Values represent means ± *SD*. Means within a column with the same lowercase letters are not significantly different at *p* < .05, and means within a row with the same uppercase letters are not significantly different at *p* < .05.

^a^ Control: cucumbers without coating.

### 3.5 TPC and YMC analysis of treated cucumbers

**FIGURE 3:**
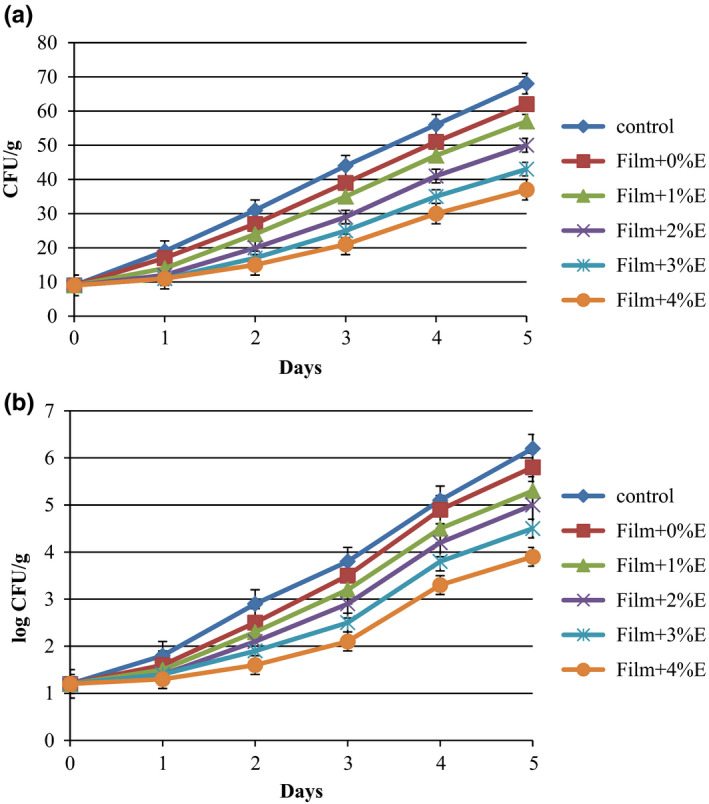
Fungal growth (a) and mesophilic microbial growth (b) on sliced cucumber coated with Konjac glucomannan + saffron petal extract during storage at 4°C. Error bars indicate the standard deviation of the CFU/g and log(CFU/g) for each film during 5 days of measurement

According to Figure 3, the YMC and TPC changes for uncoated and coated cucumber slices were determined at 4°C for 5 days. The initial counts of fungi and mesophilic bacteria were 9 ± 0.03 CFU/g and 1.2 ± 0.05 log CFU/g, respectively, which significantly (*p* < .05) increased during keeping at refrigerator for all treatments. The protective effect of KGM films was approximately low although fungi and mesophilic bacteria growth was inhibited notably as compared with control sample. As shown in Figure 3, the addition of SPE decreased the counts of both fungi and mesophilic bacteria. According to Figure 3a, an increasing trend for fungal counts was observed over time for all coated cucumbers (*p* < .05), and notable impact of SPE concentrations on fungal counts was seen during the storage (19.3%–35.1% reduction in fungal count at day 5 in comparison with control sample). Mesophilic bacteria count of control samples was recorded 5.8 log CFU/g at day 5, but for the treated cucumber slices with KGM coating containing 1%, 2%, 3%, and 4%, SPE showed noticeable reduction of 16.9%, 24.0%, 37.8%, and 58.9%, respectively, in comparison with control sample at day 5 of storage (Figure 3b). The results indicate that SPE had a significant effect on the inhibition of mesophilic bacteria growth, leading to a longer shelf life of sliced cucumbers. The results reflect that all of the treatments did not exceed the limits for mesophilic bacterial count, indicating the samples were acceptable for consumption, since the mesophilic bacterial count for food should not be higher than 6.0 log CFU/g which is specified by Institute of Food Science and Technology (Mritunjay & Kumar, 2017). Applying edible coating to the fruits and vegetables not only provides a protective layer, but also protects them against pathogenic bacteria, and also by incorporation of antimicrobial compounds such as plant extracts, this property is improved (Guerreiro, Gago, Faleiro, Miguel, & Antunes, 2015). Similar achievement was reported by Hashemi et al. (2017). On the contrary, Azarakhsh, Osman, Ghazali, Tan, and Adzahan (2014) found that alginate‐based edible coating did not reduce the mesophilic bacterial and fungal counts of fresh‐cut pineapple during the storage at 10°C.

### 3.6 Total soluble solids of treated cucumbers

**FIGURE 4:**
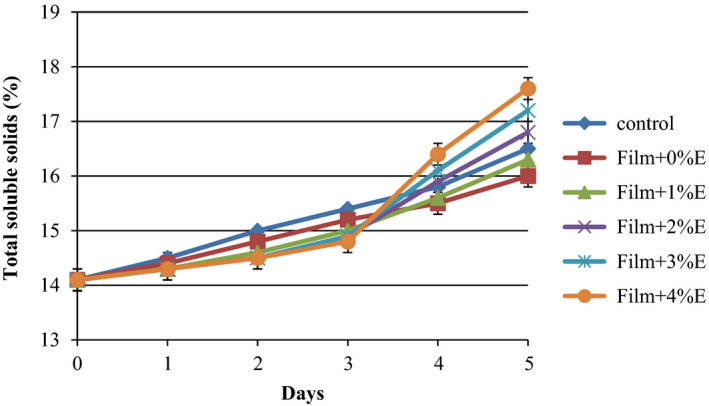
Total soluble solids of sliced cucumber coated with Konjac glucomannan + saffron petal extract during 5 days of storage at 4°C. Error bars indicate the standard deviation of the total soluble solids for each film during 5 days of measurement

Total soluble solid (TSS) contents of the various cucumber cuts during the storage period at refrigerator are shown in Figure 4. Results revealed a gradual increase in TSS for all tested groups until the 3rd day of storage which is related to the progress of sample ripening. For control cucumbers, data demonstrated that TSS amounts were about 14.1% which increased to 16.5% at the end of the storage period. After 3 days, the control samples had markedly (*p* < .05) higher TSS content (15.4%) than coated cucumbers with KGM film. Our result agrees with the report of Tien, Vachon, Mateescu, and Lacroix (2001) that they suggested protein coating to minimize the alterations in postharvest physicochemical properties, like TSS content. On the fifth day of storage, a significant spread of TSS content was observed for the different cucumber slices, with the lowest amounts being those of without coating and coating with pure KGM, followed by samples coated with KGM + SPE (1%–4%). Perdones, Sánchez‐González, Chiralt, and Vargas (2012) reported that chitosan–lemon essential oil coatings had a slight increase in the total soluble solid content, while control showed a decrease in TSS. The increase observed in the total soluble solid content during storage period might be due to the metabolism of acid and converting acids and starch into simple sugars as a consequence of ripening and senescence. Generally, sugars are the primary ingredients of TSS content of the product which are metabolized during respiration (Yaman & Bayoindirli, 2002).

### 3.7 Antioxidant activity and total soluble phenolics of treated cucumbers

**FIGURE 5:**
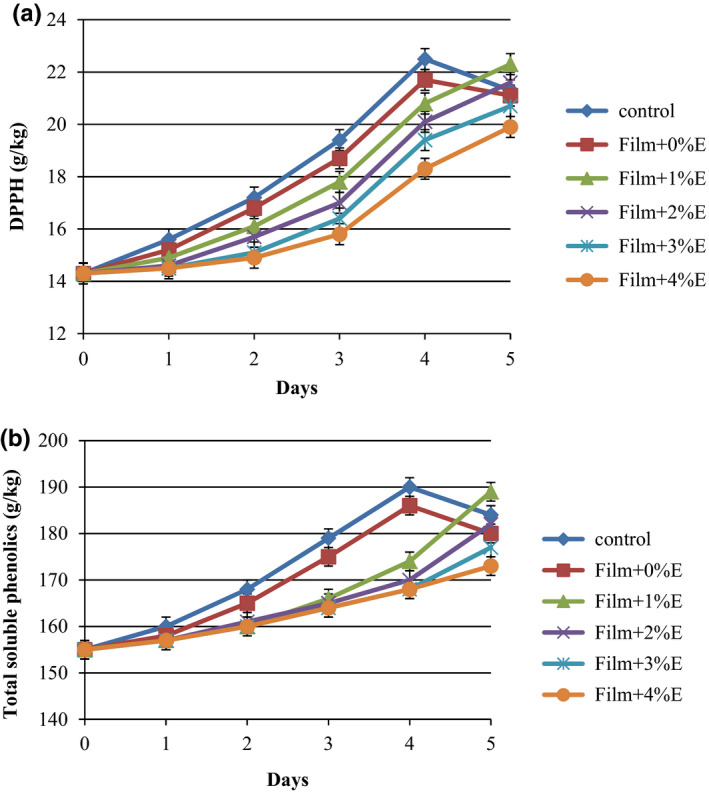
Antioxidant capacity (a) and total soluble phenolics (b) of cucumber slices coated with Konjac glucomannan + saffron petal extract during 5 days of storage at 4°C. Error bars indicate the standard deviation of the DPPH and the total soluble phenolics for each film during 5 days of measurement

The alternations in the DPPH radical scavenging activity of treated cucumbers are shown in Figure 5 A. Control sample and KGM‐coated sliced cucumbers showed the highest DPPH scavenging activity among all treatments during first 4 days of storage (22.5 g/kg), although a slight reduction was recorded until the end of the storage. Also, it can be seen that sliced cucumbers coated with SPE incorporated into KGM showed an increment in DPPH radical scavenging activity during 5 days of storage (Figure 5a). Similar results are reported for strawberries treated with ethanol vapors and methyl jasmonate (Aiala‐Zavala, Wang, Wang, & Gozales‐Aguilar, 2005). Previous research has shown that edible coating can act as a good barrier to reduce oxygen intake and also decrease oxidative degradation of antioxidant compounds (Guerreiro et al., 2015). The antioxidant activity of sliced cucumbers coated with KGM and different concentrations of SPE is also evident in the total soluble phenolics (TSP; Figure 5b). With regard to the DPPH radical scavenging activity of cucumbers, the same trend was observed for TSP, too. Cucumbers without and with KGM coating illustrated a sharp increase up to the fourth day of storage and then a gradual reduction at the end of the storage. Further, TSP content in treated cucumbers with SPE‐incorporated KGM slowly increased up to the 4th day and afterward quickly in the last day of storage (11.61%–21.93% depending on the SPE concentration). Also, it can be said that the amount of total soluble phenols in treated cucumbers decreased as the concentration of SPE‐incorporated KGM increased. Phenols are important antioxidants in fruit and plant tissues which are produced under abiotic and biotic stress conditions to preserve cellular components (Cisneros‐Zevallos, 2003). Hence, in treated cucumbers, SPE‐incorporated KGM coatings were able to prevent stress well until day 4 but the TSP significantly increased on the last day due to the possible stresses such as constant exposure of cucumbers to antimicrobial compounds in SPE, fruit senescence, and breakdown of cell structure as part of senescence (Fagundes, Carciofi, & Monteiro, 2013). Similarly, Peretto et al. (2014) demonstrated that carvacrol and methyl cinnamate antimicrobial vapors released from edible film significantly decreased the TSP of strawberry during exposure.

## 4 CONCLUSIONS

The addition of the saffron petal extract to Konjac glucomannan biodegradable film led to the production of edible film with promising antioxidant and antimicrobial properties. Furthermore, saffron petal extract markedly improved the physical characteristics (transparency, moisture content, and water vapor permeability) of produced film. According to our findings, Konjac glucomannan film with saffron petal extract prolonged the shelf life of sliced cucumbers as a result of decreasing the spoilage of them and keeping the quality features of cucumbers. The obtained results can be used for industrial applications because the proposed film can be a good alternative to synthetic packagings. However, more researches are needed for investigating the potential of the produced film in the food preservation.

## CONFLICT OF INTEREST

The authors declare no potential conflicts of interest concerning the research, authorship, and publication of this article**.**

## AUTHOR CONTRIBUTION

Seyed Mohammad Bagher Hashemi and Dornoush Jafarpour conceived of the presented idea. Seyed Mohammad Bagher Hashemi and Dornoush Jafarpour carried out the experiment and wrote the manuscript.

## ETHICAL APPROVAL

The human and animal testing was unnecessary in the current study.
